# Predation by avian predators may have initiated the evolution of myrmecomorph spiders

**DOI:** 10.1038/s41598-021-96737-2

**Published:** 2021-08-26

**Authors:** Petr Veselý, Juraj Dobrovodský, Roman Fuchs

**Affiliations:** Faculty of Science, University of South Bohemia, Branišovská 1760, 37005 Ceske Budejovice, Czech Republic

**Keywords:** Behavioural ecology, Evolutionary ecology, Evolution, Zoology

## Abstract

Myrmecomorphy is a strategy utilized by a variety of species, among which spiders are the most common. It is supposed that myrmecomorphy tends to be selected by predator avoidance of preying on ants rather than by blind ant workers. To date, this hypothesis has been tested mainly on invertebrate predators (mantises and spiders). We are the first to test whether an imperfect myrmecomorph spider (*Phrurolithus festivus*) gains protection against avian predators (wild adult great tits—*Parus major*) through its appearance. In a set of preferential trials, we showed that the ant model and the myrmecomorph spider are equally well protected against attack, though the attacked myrmecomorphs are usually eaten. This suggests that the mimicry of the myrmecomorph spiders is effective against avian predators and works in a Batesian manner. In this study, we have provided evidence toward the evolution of myrmecomorphy in response to selective pressure elicited by visually-oriented predators like birds.

## Introduction

**Figure 1:**
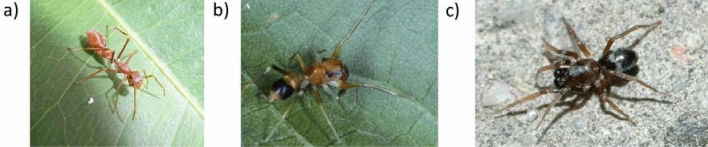
(**a**) Adult female of *Myrmarachne assimilis* ©Takeshi Yamasaki^43^ with permission; (**b**) adult female of Myrmarachne bakeri ©Robert R. Jackson^44^ with permission; (**c**) adult male of Phrurolithus festivus ©Rudolf Macek (www.pavouci-cz.eu).

Myrmecomorphy is a specific type of visual mimicry residing in the visual resemblance of an animal to an ant^1^. Spiders are common myrmecomorphs, with myrmecomorph species occurring in 13 families with most species in the family Salticidae^2^. The resemblance to the ant model may vary in perfection. There are some stunning examples of perfect resemblance, especially within a predominately tropical genus of salticid spiders, *Myrmarachne* (Aranea, Salticidae; Fig. 1a). These spiders co-occur with ants, they build their nest close to the ant nests and encounter them daily^3^. Aggressive interactions between them are rare, as *Myrmarachne* spiders usually prey on small invertebrates and their eggs^3^ and they adopt a behaviour resembling the interspecific communication of ants to avoid being attacked by them^3^. Within the genus, the spiders display a variability in the level of similarity to their ant models, with e.g., *Myrmarachne bakeri* being seen as an imperfect mimic (^4^, Fig. 1b).

Even spiders with significantly lower levels of myrmecomorphy than *M. bakeri* are called myrmecomorphs. Their body and leg shapes differ from ants, but they may be confused with ants according to colouration and means of locomotion. *Phrurolithus festivus* (Aranea, Phrurolithidae, C. L. Koch 1835; Fig. 1c^5,6^) may be a good example. Similarly as in the genus *Myrmarachne*, it commonly forages within ant swarms and usually preys on small invertebrates flushed out by foraging ants^7^.

The evolutionary importance of myrmecomorphy in spiders is still not clear. Ants generally represent a threat to the associated spiders^8^. Therefore, their morphological resemblance used to be supposed by human observers to be an adaptive protection against being attacked by the ants. Nevertheless, ant soldiers are almost blind and cannot perceive the visual appearance of the myrmecomorphs. Thus, the ant mimicry (Wasmannian and/or Peckhamian^7,9^) securing the confusion in ants regarding myrmecomorphs is usually based on chemical or tactile cues^10–12^.

A less obvious possibility is that the visual resemblance of the myrmecomorph spiders to ants may be a signal to the former’s potential predators. This hypothesis has been tested repeatedly in perfect mimics of genus *Myrmarachne*. The predators used were mantises^4,13^ or salticid spiders^14,15^. The protection of *Myrmarachne* species against all of the invertebrate predators was very good when compared with non-myrmecomorph salticid spiders.

The only imperfect mimic used in these studies was *Myrmarachne bakeri* (Fig. 1b). Mantises showed a slightly higher willingness to prey on them when compared to perfect mimics^4^, though its protection can be still considered as very effective, when compared to non-myrmecomorph salticids. The protection of species with less perfect resemblance to ants against predators has been tested experimentally only scarcely and never in the genus Phrurolithus (Fig. 1c). We can suppose that when inspected from close proximity (by mantises or spiders), such myrmecomorphs may be distinguished from the ants more easily than perfect *Myrmarachne* mimics. Regardless, there are also other visually oriented predators, which usually do not have this opportunity to inspect the small invertebrate prey in detail: birds.

Birds are usually considered to be the main selective force forming visual mimicry in invertebrates^16–18^. We can expect that imperfect myrmecomorphy in spiders is also selected by avian predators. Foraging birds must usually identify prey from a distance of tens of centimetres. Moreover, when encountering an ant swarm, even imperfect mimics may be avoided due to the decreased identification abilities at such a range^19^. The experimental evidence for the effective function of ant mimicry as a protection against birds is lacking. Most European insectivorous birds collect ants; nevertheless, the proportion of ants in their diet is usually very low^20–23^. With the exception of a few specialized species (e.g. woodpeckers) ants are not the preferred prey of insectivorous birds and ants may thus act as models in this mimicry system.

In the present study, we tested the level of protection of the imperfect myrmecomorph spider *Phrurolithus festivus* against an insectivorous avian predator, the great tit (*Parus major* L. 1758). We simultaneously presented wild caught great tits with the following pairs of prey: myrmecomorph spider (*Phrurolithus*) and ant (*Lasius niger* L. 1758—hereafter called *Lasius*); myrmecomorph spider and non-myrmecomorph wolf spider (*Alopecosa*); and ant together with Mediterranean cricket (*Gryllus bimaculatus*, De Geer 1773, *Gryllus*) as a control for the avoidance of ants. We also recorded the total time birds observed the prey from a distance (i.e. a proxy for attention paid to the pair of prey). This behaviour was understood as a measure of hesitation before the attack in previous studies^24,25^.

We tested the following hypotheses:

1. Tits attack and eat the ants less than an edible insect prey (cricket)—ants can act as models in the evolution of myrmecomorphy selected by avian predators.
2. Tits attack the myrmecomorph spiders equally as often as the ants and less often than the non-myrmecomorph spiders—myrmecomorphy is a successful form of mimicry in the evasion of avian predators.
3. The attacked myrmecomorph spiders are eaten by tits more often than the ants and equally often as the non-myrmecomorph spiders—the myrmecomorph mimicry works in a Batesian manner.

## Results

### Observing the prey from distance

**Figure 2:**
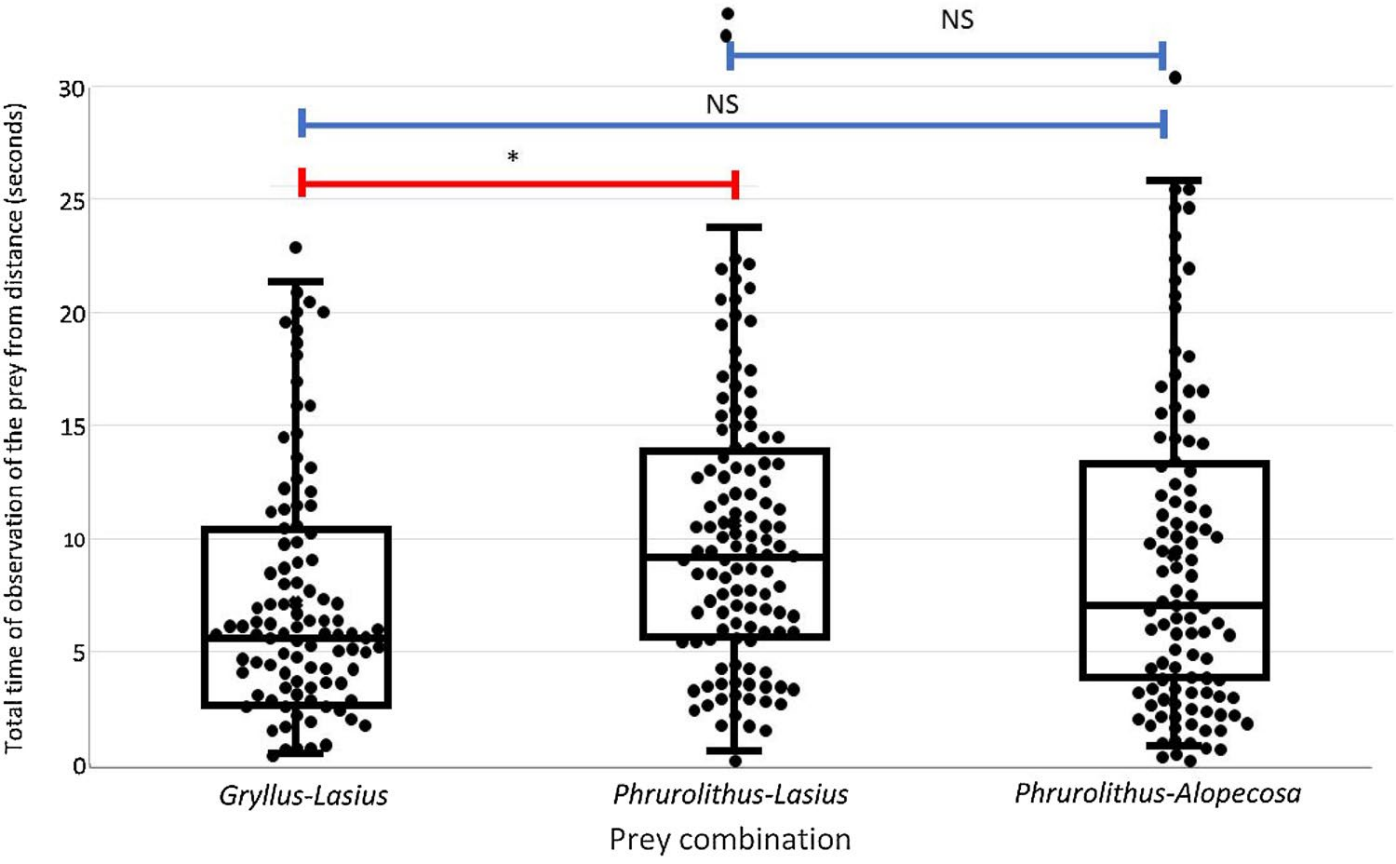
The total time spent observing the pair of prey offered simultaneously. *Gryllus*—Mediterranean cricket (*Gryllus bimaculatus*), *Lasius*—black garden ant (*Lasius niger*), *Phrurolithus*—myrmecomorph spider (*Phrurolithus festivus*), *Alopecosa*—non-myrmecomorph wolf spider of genus *Alopecosa*. Median marked with horizontal line, box refers to 25–75% quartiles, whiskers 10–90% quantiles. All data represented by dots. Red lines with an asterisk above the columns indicate significant difference, blue lines indicate non-significant difference (NS)^42^.

The total time the birds spent observing a particular pair of offered prey items from a distance can be seen as a measure of hesitation whether to attack the prey or not. We showed that it was significantly affected by the particular combination of presented prey (Table 1). Great tits observed the *Phrurolithus*–*Lasius* combination for the longest time and the *Gryllus*–*Lasius* combination for the shortest time (Fig. 2), with the difference between them being significant (Tukey HSD post hoc test, Z = 3.099, P = 0.006). *Phrurolithus*–*Alopecosa* combination was observed for a shorter time than *Phrurolithus*–*Lasius* and for a longer time than *Gryllus*–*Lasius*, but none of these comparisons were significant (Tukey HSD post hoc test, Z = 1.246, P = 0.426 and Z = 1.812, P = 0.166 respectively).

**Table 1:** Effects of tested predictors on behaviour of avian predators (Generalized or Linear Mixed-effect Models, likelihood ratio tests).

Behavioural response	Predictor	Chi-square	DF	P
Observing the prey from distance	**Prey combination**	**9.401**	**2**	**0.009**
	Trial × prey	7.256	14	0.411
Attacking the prey	**Type of the prey**	**182.12**	**5**	**<< 0.001**
	Trial × prey	11.256	24	0.328
Eating the prey (only attacked prey)	**Type of the prey**	**99.831**	**5**	**<< 0.001**
	Trial × prey	11.896	24	0.227

*DF* degrees of freedom. Significant effects in bold.

### Attacking

**Figure 3:**
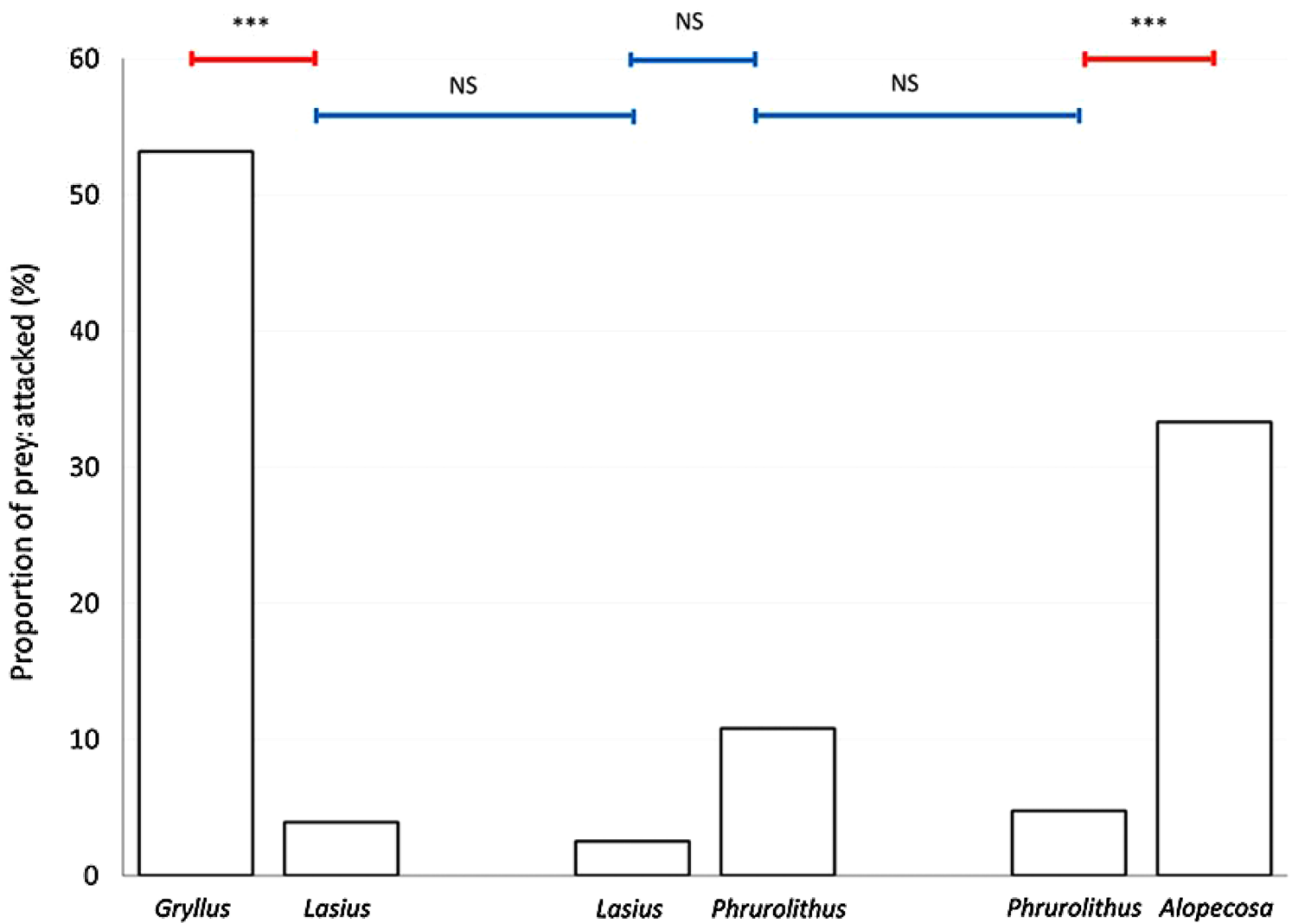
The proportion of attacked offered prey in particular prey pairs. *Gryllus*—Mediterranean cricket (*Gryllus bimaculatus*), *Lasius*—black garden ant (*Lasius niger*), *Phrurolithus*—myrmecomorph spider (*Phrurolithus festivus*), *Alopecosa*—non-myrmecomorph wolf spider of genus *Alopecosa*. Red lines with an asterisk above the columns indicate significant difference, blue lines indicate non-significant difference (NS)^42^.

The attacking of prey indicates a lack of visual avoidance since the birds will be unlikely to perceive any chemosensory differences at this point. The number of prey attacked was significantly affected by the type of prey (Table 1). Ants were attacked significantly less than crickets (Fisher LSD post hoc test, Z = 11.775, P < 0.001; Fig. 3). Myrmecomorph spiders were attacked less often than non-myrmecomorph spiders (Fisher LSD post hoc test, Z = 7.035, P < 0.001; Fig. 3), and equally often as the ants (Fisher LSD post hoc test, Z = 2.194, P = 0.221; Fig. 3). Ants presented together with crickets were attacked equally often as ants presented together with myrmecomorph spiders (Fisher LSD post hoc test, Z = 0.240, P = 0.999; Fig. 3). Myrmecomorph spiders presented together with ants were attacked equally often as myrmecomorph spiders presented together with non-myrmecomorph spiders (Fisher LSD post hoc test, Z = 0.986, P = 0.914; Fig. 3).

### Eating of attacked prey

**Figure 4:**
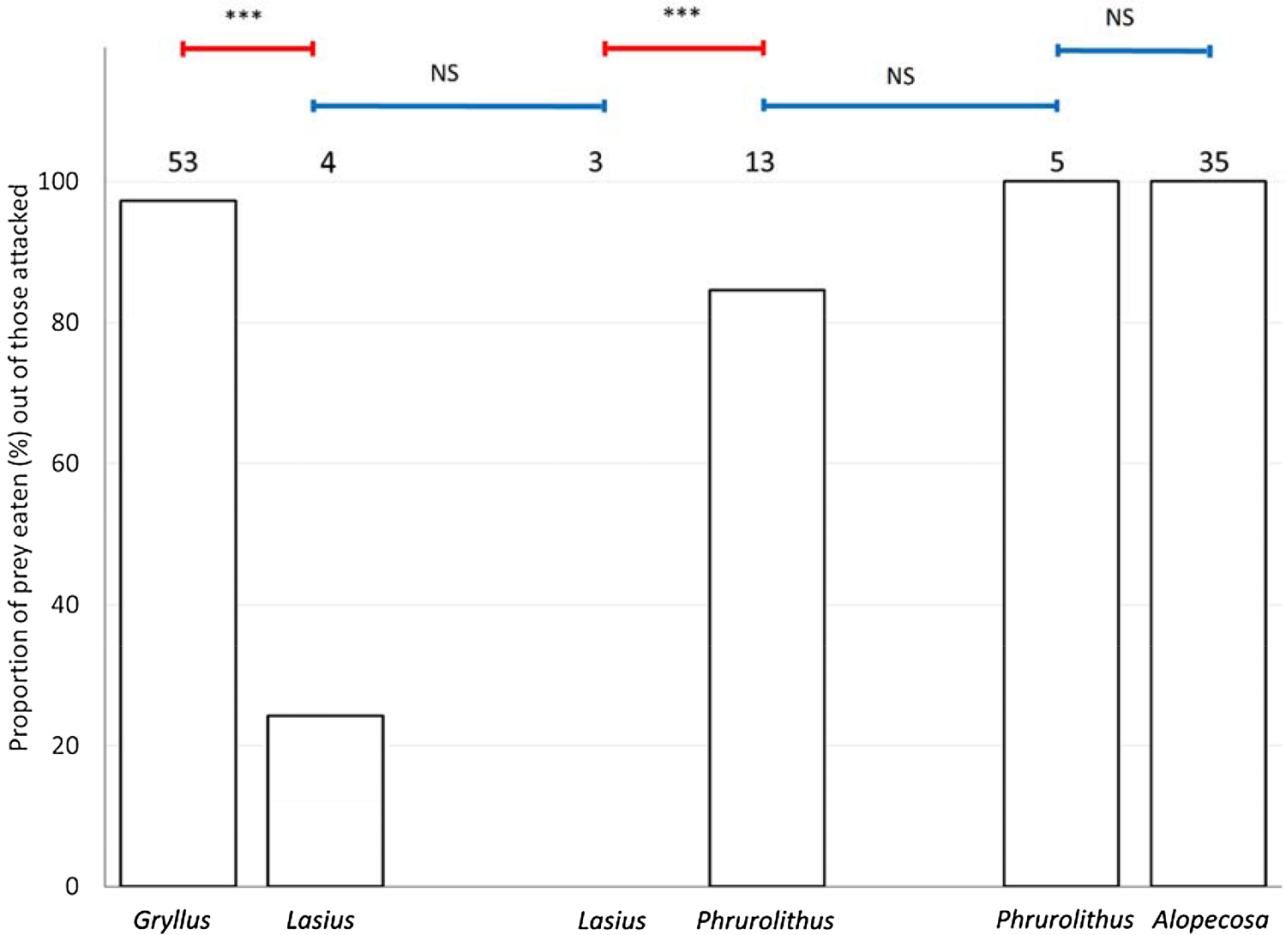
The proportion of particular prey being eaten after the attack. *Gryllus*—Mediterranean cricket (*Gryllus bimaculatus*), *Lasius*—black garden ant (*Lasius niger*), *Phrurolithus*—myrmecomorph spider (*Phrurolithus festivus*), *Alopecosa*—non-myrmecomorph wolf spider of genus *Alopecosa*. The numbers above each column refer to the number of particular prey being attacked and thus included in this analysis. Red lines with an asterisk above the columns indicate significant difference, blue lines indicate non-significant difference (NS)^42^.

Eating was scored in a situation when the attacked prey was at least partially eaten (usually it was completely eaten). It can be seen as an effect of the chemical composition of the prey on the bird, which may or may not eat the prey following the initial attack. The number of eaten prey, out of those attacked, was significantly affected by the type of prey (Table 1). Ants were eaten significantly less than crickets (Fisher LSD post hoc test, Z = 7.883, P < 0.001; Fig. 4) and myrmecomorph spiders (Fisher LSD post hoc test, Z = 7.386, P < 0.001; Fig. 4). Myrmecomorph spiders were eaten equally often as the non-myrmecomorph spiders (Fisher LSD post hoc test, Z = 0.001, P = 0.999; Fig. 4). Ants presented together with crickets were eaten equally often as ants presented together with myrmecomorph spiders (Fisher LSD post hoc test, Z = 1.830, P = 0.411; Fig. 4). Myrmecomorph spiders presented together with ants were eaten equally often as myrmecomorph spiders presented together with non-myrmecomorph spiders (Fisher LSD post hoc test, Z = 1.634, P = 0.541; Fig. 4).

## Discussion

Ants were attacked by great tits less than the larval instars of crickets of the same size. They may thus probably act as protected models in the evolution of myrmecomorphy selected by avian predators. The great tits attacked the myrmecomorph spiders less than the non-myrmecomorph spiders and equally often as the ants. The visual appearance of myrmecomorph spiders provides an equal level of protection against the insectivorous passerine, the great tit, as the ants possess themselves. We thus experimentally confirmed that the imperfect myrmecomorph *Phrurolithus festivus* is effectively protected against bird predators. The attacked myrmecomorph spiders were eaten very often, significantly more often than the attacked ants and equally often as the attacked non-myrmecomorph spiders. We may thus suggest that myrmecomorph spiders possess no chemicals acting as repellents to avian predators and can thus be treated as Batesian ant mimics. Ants possessing formic acid (see below) act thus as models avoided by birds in this system.

Our results suggest that the visual signals of at least some ant-mimicking invertebrates may be addressed to avian predators. There are substantial differences in the vision of invertebrates and birds^26^; nevertheless, our experiments suggest that the ant-mimicking strategy has a similar effect on birds as was shown by previous studies on invertebrate predators. Experiments with skinks (Squamata, Scincidae), which possess a similar visual acuity to birds, also exhibited aversion towards myrmecomorph spiders^27^. Some studies have shown that the same appearance of myrmecomorphs may affect both visually as well as chemically oriented predators. e.g. sphecid wasps show reduced predation on myrmecomorph spiders^28^. The authors presumed that the main cue for identification of the prey in these wasps is the chemical structure of its surface; nevertheless, the body shape also secured protection for the myrmecomorph spiders, despite their chemical compounds being equal to those in most spiders. Nonetheless, further investigations with multiple predators are needed to better understand the myrmecomorph mimicry system and its evolution.

All of the above-mentioned studies confirming the efficacy of ant-mimicry on invertebrate predators used accurate mimics of ants, spiders of the genus *Myrmarachne*. It is hard to differentiate these spiders from ants even during close inspection by the human eye. Our results show that birds may be confused even by imperfect mimics. *Phrurolithus festivus* has no body and leg shape adaptation to resemble ants, they merely possess a similar manner of movement and colouration creating the impression of an ant body shape. This makes them imperfect mimics of ants. Despite this imperfection, the mimicry provides good protection against at least some avian predators. The reason may reside in the different hunting strategies of invertebrates and birds. We can presume that mantises and jumping spiders encounter the ant mimics from a close distance and perceive their visual appearance from a different perspective than birds searching for prey from larger distances. Even in our experiments, the great tits decided which prey to attack from a perch at a distance of 30 cm. Many myrmecomorphs appear within ant swarms and the aggregation secures the protection of all members of the swarm^19^. Other imperfect mimics participating in ant swarms may gain protection through this effect^29^. At first sight, our experiments also suggest the importance of this effect. When we presented a myrmecomorph spider together with an ant, the birds spent a long time observing the prey from a distance. We understand this behaviour as the bird deciding whether to attack the prey or not. Obviously, birds were a little bit confused when challenged by the recognition between the myrmecomorph and the ant. On the other hand, the great tits did not attack the myrmecomorph even when presented together with a non-myrmecomorph spider. This suggests that the visual protection of myrmecomorphs is effective regardless of the circumstances and the effect of confusion within an aggregated swarm may not play any role. The method of recognition of prey by predators will of course differ between different predators.

Our experiments showed that the attacked myrmecomorph spiders were often eaten. This was very different from ants, which were never eaten in our experiments. Ants possess a chemical protection, formic acid, which originally acts as an alarm signal but also has an antipredatory effect^30^. This obviously repels most birds that decide to attack ants. Great tits usually do not prey on ants^31,32^ and our results suggest that the main reason is their chemical protection. At the same time, myrmecomorph spiders obviously possess no chemical protection against avian predators. Once the bird attacked myrmecomorph, it was not eaten in only one of 20 trials. *Phrurolithus festivus* can be thus treated as a Batesian mimic of ants with respect to avian predators.

Our study was also the first to experimentally confirm that great tits avoid attacking ants. As mentioned above, ants do not occur in their natural diet. Most of the great tits in our experiments were able to avoid attacking the ant only according to its visual appearance. Those tits which decided to attack were further repelled by the chemical signals or cues. Most inedible prey advertise their inedibility with bright colours and patterns^33^, while ants are usually uniformly blackish, brownish, or rufous. It is obvious that the typical body, leg, and antennae shape and manner of locomotion are the visual signals enabling the proper recognition of the ant. The ability to recognize ants according to their shape and size has previously been shown in invertebrates only. Sendoya et al.^34^ showed that *Eunica* butterflies (Hübner, 1819) avoid oviposition on plants artificially associated with ant dummies. Nelson et al.^4^ also showed that mantises avoid attacking ants based on their specific visual appearance. Our results with birds thus concur with these conclusions.

To conclude, we showed that birds, as visually oriented predators, may drive the evolution of myrmecomorphy in spiders. It is likely that the array of predators, to which the myrmecomorphs address their visual signals may be much broader and many more experiments are required to broaden our understanding. The signalling of myrmecomorph spiders is much more complex, potentially encompassing not only vision, but also tactile perception, olfaction, and taste. Still, our study brings a novel view on the understanding of this spectacular adaptation, which may importantly affect future research.

## Methods

### Prey

*Phrurolithus festivus* is an imperfect myrmecomorph spider commonly occurring in Europe. It preserves a typical spider body shape but its colouration (shiny black opisthosoma with white transversal stripe and reddish-brown prosoma with median white stripe) creates the visual impression of an ant (^6^, Fig. 1c). Moreover, its jerky manner of locomotion strengthens the impression of an ant. The body size ranges from 2.2 to 3.2 mm. It can often be found within foraging ant swarms, but it does not prey on ants, and the ants also do not attack it^5^.

We compared the responses of avian predators to myrmecomorph spiders with the responses to its presumed model, the black garden ant, which possesses a similar colouration and body size. As the control non-myrmecomorph prey we used juvenile (2–3 mm in length) wolf spiders of the genus *Alopecosa,* probably *A. taeniata* (C. L. Koch 1835) or *A. pulverulenta* (Clerck 1757), it was impossible to determine the species in juveniles. These are common spiders at our localities, and we may expect tested great tits to be familiar with them as a common and palatable prey^27,28^. A baseline prey was represented by juveniles of the Mediterranean cricket, which are commonly accepted as artificial insect prey by great tits^35^.

The prey was collected in the surroundings of the town of České Budějovice from July to December a few days prior to the experiment (myrmecomorph, ant, non-myrmecomorph) or originated from commercial breeding (crickets) and were kept at low temperature (10 °C), under a 12:12 h daylight regime with moist soil provided to keep them in a diapause status until the experiment (usually only a few days). The myrmecomorph spiders were collected together with the ants used for experiments within the same swarms.

### Predators

As the avian predator we used the great tit, which is a predominately insectivorous passerine bird of medium body size (body weight 15–20 g^36^). Its diet consists especially of caterpillars, spiders, and beetles, with ants being only seldom preyed upon^27,28,37^. Previous studies have shown that great tits avoid the aposematic insects^38^, being usually discouraged by the colouration of the prey^39,40^ and by the combination of colours forming specific patterns^24,41^. Nevertheless, the attitude of great tits to ants has not been previously tested experimentally.

The birds used for the experiments were caught in the surroundings of České Budějovice, transported in commercially sold bird cages (40 × 40 × 60 cm) with water, sunflower seeds, and mealworms (larvae of *Tenebrio molitor* L. 1758) provided ad libitum, under a 16:8 daylight regime, and a 20 °C ambient temperature. Birds were individually marked with ornithological rings and each individual bird was subjected to a single experiment only.

### Experimental design

Experiments were conducted during the non-breeding season of great tits (July to December in the years 2015, 2016 and 2017), in the morning hours, when the birds were motivated to forage. The presentation of the tested prey was conducted in the experimental cage (70 × 70 × 70 cm) made of a wooden frame covered with fine wire mesh. The cage was equipped with a front wall made of one-way glass, which allowed the human observer to sit by the cage without being noticed by the bird. At the bottom of this wall, there was a rotating circular tray with eight white cups (6 cm in diameter), in which the prey was offered. There were always two cups containing a single prey item in each of them, both prey were available to the bird, and both prey could be attacked.

Once the bird was trained to accept mealworms in this cage, it was deprived of food for two hours to improve its motivation and after this period, the experiment started. The experiment consisted of five successive presentations of a pair of experimental prey items (see below for combinations) alternated with offering a small mealworm (8 mm) to check the bird’s motivation to forage. Each prey was presented in separate white cups, but both prey items were in view of a perching bird at the same time. The bird behaviour during the presence of the experimental prey in the cage was always recorded for five minutes.

Each prey combination was offered to each set of birds. We offered three pairs of prey items: (1) *Gryllus*–*Lasius*—100 prey pairs offered to 20 bird individuals; (2) *Lasius*–*Phrurolithus*—120 prey pairs offered to 24 bird individuals; and (3) *Alopecosa*-*Phrurolithus*—105 prey pairs offered to 21 bird individuals. Altogether, we used 65 bird individuals in our experiments.

### Data analyses

We evaluated three behaviours displayed by the tested birds.

We firstly recorded the total time (in seconds), that the tested bird spent observing the pair of prey items from a distance. We were not able to differentiate at which of the two offered prey items the bird was looking; therefore, we evaluated the total time of observation for the entire pair. These data followed the gaussian distribution of residuals, therefore we used a linear mixed effect model (command lmer in R package lme4^42^) to evaluate the effect of predictors on the variability in this data type. We used a mixed effect model because five prey pairs were always offered to the same bird, thus the bird ID was included in the model as the random factor. We tested the effect of two predictors: the prey combination (*Gryllus*–*Lasius*, *Lasius*–*Phrurolithus*, *Alopecosa*–*Phrurolithus*) and the interaction of prey combination and the trial number (coded as categorical predictor with five values—first to fifth). We used likelihood ratio tests for the Gaussian distribution of data (Chi squared test) to compare the models in the forward stepwise selection. For the comparison of particular prey types, we used Tukey HSD post hoc tests (Z test) with Tukey correction for repeated comparisons.

The second evaluated behaviour of the tested birds was the occurrence of the attack to the prey, which was recorded once the bird pecked the prey or took the prey into its bill. This response variable scored 1, when the prey was attacked and 0 when it was not. The effect of predictor variable prey type and the interaction of prey type and trial order (both categorically coded) was evaluated using generalized mixed-effect linear model following binomial distribution with bird ID as a random factor (command glmer in R package lme4^42^). We used likelihood ratio tests for the binomial distribution of data (Chi squared test) to compare the models in the forward stepwise selection. For the comparison of the prey, we used Fisher LSD post hoc tests (Z test) with Tukey correction for repeated comparisons.

The third behaviour of tested birds evaluated how often prey were eaten once they had been attacked, therefore only trials in which the attack occurred were included in this analysis. The eating of the prey was scored in a situation when the bird consumed at least part of the prey item’s body. The prey items were small compared to the great tits; therefore, the tested birds usually ate the entire prey item. The effect of predictor variable prey type and the interaction of prey type and trial order on the binomially coded occurrence of eating was evaluated using generalized mixed-effect linear model following binomial distribution with bird ID as a random factor (command glmer in R package lme4^42^). We used likelihood ratio tests for the binomial distribution of data (Chi squared test) to compare the models in the forward stepwise selection. For the comparison of the prey types, we used Fisher LSD post hoc tests (Z test) with Tukey correction for repeated comparisons.

### Ethics approval

All applicable international, national, and/or institutional guidelines for the care and use of animals were followed. Experimental birds were kept in accredited breeding of birds at the Faculty of Science, University of South Bohemia (permit no. 22395/2014-MZE-17214 issued by the Ministry of Agriculture of the Czech Republic). Permission for studies on wild great tits was granted by the Ministry of the Environment of the Czech Republic (permit no. 18232/ENV/15-833/630/15). The study was approved by the ethics committee of the Faculty of Science, University of South Bohemia. The authors are licenced for experimentation with animals (PV-CZ02766, RF-CZ01629, licences issued by the Ministry of the Agriculture of the Czech Republic). This research adhered to the ASAB/ABS guidelines for the use of animals in research. Authors declare that the experiments comply with the current laws of the Czech Republic (and European Union).

## Supplementary Information

Supplementary Legends.

Supplementary Table S1.

## Data Availability

The original data sheet is available as the supplementary information to this manuscript.
