# Supplementary material for: Predation by avian predators may have initiated the evolution of myrmecomorph spiders
Source: Sci Rep. 2021 Aug 26;11:17266. doi: 10.1038/s41598-021-96737-2 (PMC8390495; doi:10.1038/s41598-021-96737-2)
Supplement: Supplementary file 1 — Supplementary Legends. [file 41598_2021_96737_MOESM1_ESM.docx]

**Supplementary information**

**Table S1** – The data sheet used for the analyses performed in this paper.
